# Evaluation of Productivity and Egg Quality in Japanese Quails Reared Under Different LED Colors and Rearing Systems

**DOI:** 10.3390/vetsci13020164

**Published:** 2026-02-07

**Authors:** Paitoon Kaewhom, Kraiyot Saelim, Patcharawadee Poolsamran, Chanathip Thammakarn, Chanporn Chaosap, Rasheed Olayiwola Sulaimon, Panneepa Sivapirunthep, Kanokrat Srikijkasemwat

**Affiliations:** 1 Faculty of Agricultural Technology, Burapha University, Sakaeo Campus, Sa Kaeo 27160, Thailand; paitoon_k@buu.ac.th (P.K.); kriyot@buu.ac.th (K.S.); 2 Department of Mathematics, Faculty of Science, Burapha University, Chon Buri 20131, Thailand; patcharp@buu.ac.th; 3 Department of Animal Production Technology and Fisheries, School of Agricultural Technology, King Mongkut’s Institute of Technology Ladkrabang, Bangkok 10520, Thailand; chanathip.th@kmitl.ac.th; 4 Department of Agricultural Education, School of Industrial Education and Technology, King Mongkut’s Institute of Technology, Ladkrabang Bangkok 10520, Thailand; chanporn.ch@kmitl.ac.th (C.C.); 67036072@kmitl.ac.th (R.O.S.); panneepa.si@kmitl.ac.th (P.S.); 5 Department of Animal Science, Faculty of Agriculture, University of Abuja, Abuja 902101, Nigeria

**Keywords:** production performance, laying performance, egg production, photo stimulation, avian reproduction

## Abstract

This study evaluated the productivity and egg quality of Japanese quails reared under different LED colors and rearing systems. A total of 720 female quails were assigned to a 3 × 2 factorial arrangement with three LED colors (red, green, and white) and two rearing systems (cage and floor) until 20 weeks of age. Production performance was evaluated across specific age intervals, while physical egg quality traits were analyzed using a Repeated-measures General Linear Model to assess temporal changes. No significant overall interactions between LED color and rearing system were observed (*p* > 0.05). However, significant interactions between treatment and time (*p* < 0.05) revealed that red LED light progressively enhanced productivity, while the floor system significantly improved feed efficiency and income during the early laying phase (weeks 6–12). Specifically, red LED light significantly improved hen-day production, egg mass, feed efficiency, and income-to-cost ratio compared to other colors (*p* < 0.05). Physical egg quality traits remained consistent across treatments (*p* > 0.05) but were significantly influenced by time (*p* < 0.05). In conclusion, red LED light optimizes long-term profitability, whereas the floor system offers distinct advantages during the onset of lay.

## Simple Summary

Proper lighting and housing are key to success in poultry farming, yet the best combination for quail production is often debated. We conducted this study to see how different LED light colors, red, green, and white, alongside two rearing systems, cages and floor pens, affect how Japanese quails grow and lay eggs. Our results revealed that while the choice of housing showed similar long-term results, the floor system was more efficient during the early laying stage. The color of the light was very important. Quails raised under red LED lights performed better, laying more eggs and using their feed more efficiently, which ultimately led to higher earnings. Notably, the physical quality of the eggs stayed the same, no matter which light color or housing was used. These findings offer a simple, practical tip for farmers: switching to red LED lighting and considering floor rearing for the initial period can boost productivity and profit without requiring expensive equipment or complex changes. This approach provides a straightforward way to make quail farming more efficient and sustainable for the industry.

## 1. Introduction

Lighting constitutes a critical environmental and exogenous regulatory factor in intensive poultry production systems. Beyond its role in visual illumination, light significantly influences physiological functions, behavioral patterns, growth dynamics, and the overall productive performance of poultry species [1,2]. In Japanese quails, the spectral composition of artificial light plays a vital role in modulating production traits and reproductive maturation [3]. Implementing well-structured lighting programs is essential for optimizing egg production, as light serves as a primary stimulus affecting avian endocrine responses. It is widely recognized to be associated with the regulation of reproductive hormone secretion, potency influencing the onset of sexual maturity and initiation of egg laying [4].

Light-emitting diodes (LEDs) are increasingly being adopted as efficient alternatives to traditional incandescent and fluorescent lighting systems in poultry housing [5,6]. This transition is driven by high energy efficiency, extended operational lifespan, spectral versatility, reduced electricity consumption, and the potential of LEDs to lower overall production costs [2,7,8,9]. Different LED light colors exert varying effects on performance parameters across various stages of the avian production cycle [10], including key egg quality indicators [1]. Similar calming effects have been observed across various poultry species [5,11,12,13], suggesting a conserved behavioral response to specific spectra. Moreover, light color significantly regulates reproductive processes in birds [14]. Bobadilla-Mendez et al. [15] reported that photostimulation using white light was more effective in initiating the reproductive cycle, advancing the onset of sexual maturity, and supporting post-pubertal development of reproductive organs of Japanese quail. Similarly, Elkomy et al. [3] found that exposure to red light during both the growth and laying periods significantly enhanced growth and reproductive performance in Japanese quails.

In addition to lighting, the rearing system is one of the most influential factors affecting poultry welfare, health, and overall production efficiency [16]. Japanese quails are typically raised in multi-tiered cages during both growth and laying periods. However, floor-based systems have also demonstrated comparable efficiency without negatively impacting egg production [17]. Badawi [18] reported no significant differences in productivity indices such as egg yield, feed conversion, and survivability between cage and floor-reared quails. Despite growing interest in the use of LED lighting in poultry production, research on Japanese quails has largely focused on either production performance or reproductive outcomes under lighting systems [3,15], with little emphasis on the potential synergistic or antagonistic interactions between different light colors and housing conditions. Furthermore, existing reports have provided inconsistent findings, with some studies showing no effect of LED colors on growth traits [19], while others reported improved laying rate and egg mass under red light [20]. Therefore, this study was undertaken to address this gap by evaluating the integrated effects and interactions of LED light colors and rearing systems on laying Japanese quails. It was hypothesized that different LED light spectra would differentially influence growth performance and reproductive output in laying Japanese quails, with green light potentially modulating reproductive activity compared with red and white light. However, responses appear to depend on species, age, housing conditions, and stage of production. Under practical rearing conditions, the extent to which specific interaction between LED colors and housing environment alters productivity remains incompletely understood. We hypothesized that exposure to red LED light, owing to its stronger physiological stimulation of the reproductive axis [14,21], may enhance egg production traits compared to white and green lights, while cage and floor systems may exert comparable effects on productivity and physical egg quality. Furthermore, we questioned whether calculating egg production from 4 to 20 weeks would reveal photoreceptor effects on the onset of laying. Therefore, the objective of this study was to evaluate the effects of different LED light colors and rearing systems on growth performance, egg production, and physical egg quality traits in laying Japanese quails, with a particular emphasis on the temporal dynamics and phase-dependent responses throughout the production cycle from the onset of lay to physiological maturity.

## 2. Materials and Methods

### 2.1. Birds, Housing, Diets, and Experimental Design

A total of 720 female Japanese laying quails (*Coturnix japonica*) were obtained from a commercial farm at 23 days of age and acclimatized for 5 days before being included in the experiment at 28 days of age. Birds were randomly assigned to treatment groups in a 3 × 2 factorial arrangement using a completely randomized design, consisting of three LED light colors (red, white, and green) and two rearing systems (cage and floor), with three replicates per treatment combination. Each treatment combination included three replicates of 40 birds, resulting in a total of 720 quails. Based on the resource equation method [22,23], the sample size was evaluated by calculating the value E as the difference between the total number of experimental units and the total number of groups, where an acceptable range is 10 to 20. In this study, a 3 × 2 factorial arrangement was used, resulting in six treatment groups, each with three replications, giving a total of 18 experimental units. Accordingly, E was 18 − 6 = 12, which lies within the recommended range; therefore, three replications per treatment combination were considered an appropriate sample size.

The housing consisted of a hierarchical enclosure system divided vertically and horizontally with a metal chain link fence covered with black opaque plastic sheeting into three housing units, each measuring 4 × 4 × 3 m (W × L × H). To ensure that only the intended LED light spectra were received by the birds, each LED treatment was assigned to a separate housing compartment enclosed with black opaque plastic curtains on all sides. These acted as light barriers, preventing external ambient light intrusion and avoiding light contamination between treatments. Light intensity (40 lux at bird head level) was verified weekly using a digital lux meter to confirm consistency. Each housing unit was assigned one of the three LED light treatments: white (400–700 nm), red (618–635 nm), or green (515–535 nm). To ensure precise lighting control, a 16-h light and 8-h dark photoperiod was maintained throughout the study using an automated timer system. The light intensity was strictly set at 40 lux at the birds’ head level, which was measured and verified weekly using a digital lux meter (SRI 2000; Shangze Photoelectric Co., Ltd., Hsinchu, Taiwan) to ensure uniform luminance across all compartments, and in accordance with luminance requirements for laying Japanese quails (20 lux) [24]. Within each housing unit, space was divided into four compartments. Three compartments were used for the floor rearing system (pen size 1 × 1.5 × 3 m, W × L × H) with a 5 cm thick layer of clean rice husk as bedding, which was managed daily to maintain a dry and hygienic environment for the birds, while the remaining compartment was used for the cage rearing system. Ventilation fans were installed within the bird house to ensure adequate air circulation across all compartments. The cage system consisted of three layers of metal cages, each measuring 1 × 1.5 × 0.5 m (W × L × H). The stocking density was maintained at 375 cm^2^ per bird for both rearing systems, ensuring optimal animal welfare. Feed was provided ad libitum using different equipment: in the cage system, linear manual troughs (10 × 120 cm) were positioned at the front of each cage, while in the floor system, hanging tube feeders were used at a ratio of one feeder per 20 birds. Clean water was accessible at all times through an automatic nipple drinking system. The rice husk bedding in the floor pens was inspected daily and managed to remain dry and hygienic throughout the study.

Birds were nourished with a commercial mash diet formulated for laying Japanese quails. According to the manufacturer’s guaranteed analysis, the diet contained a minimum of 22% crude protein and 3% crude fat, with a maximum of 5% crude fiber and 13% moisture. The primary ingredients included fish meal, soybean meal, sunflower meal, de-oiled rice bran, and leucaena leaf meal.

The trial was conducted during the rainy season (June to November) in Thailand, in open housing with natural ventilation. Ambient temperature ranged from 21.0 to 27.5 °C (mean 25.5 °C), and relative humidity averaged 89.5%.

### 2.2. Growth Performance

Growth performance data were recorded from 4 to 20 weeks of age, following the procedure described by Ahmad et al. [25]. Birds were weighed individually at the start of the experiment and on a weekly basis thereafter to monitor their growth. Body weight gain (BWG) and average daily gain (ADG) were calculated from these measurements. Feed intake was recorded by subtracting leftover feed from the total feed offered. Viability was assessed by recording mortality throughout the study and expressing it as a percentage of the initial population.

### 2.3. Egg Production

Eggs were collected daily throughout the laying period to determine cumulative hen-day egg production (HDP), egg weight (EW), egg mass (EM), feed conversion ratio (FCR; kg feed per kg egg mass), and income-to-cost ratio (IC), following standard procedures for Japanese quail [26,27]. Egg production was recorded from 6 weeks onward, corresponding to the onset of lay in Japanese quails. Individual egg weights were measured using a precision digital balance with an accuracy of 0.01 g. Feed intake per replicate was monitored concurrently to compute feed conversion efficiency per unit of egg mass. The EM and FCR were calculated using the following equations:

$$\mathrm{Egg}\;\mathrm{mass}\;(g\cdot {\mathrm{bird}}^{-1}\cdot d^{-1})=\frac{\mathrm{Hen}-\mathrm{Day}\;\mathrm{Egg}\;\mathrm{Production}\;\times \;\mathrm{Average}\;\mathrm{egg}\;\mathrm{weight}\;(g)}{100}$$

$$\mathrm{FCR}\;=\frac{\mathrm{Total}\;\mathrm{feed}\;\mathrm{intake}\;\left(\mathrm{kg}\right)}{\mathrm{Total}\;\mathrm{egg}\;\mathrm{mass}\;\mathrm{produced}\;\left(\mathrm{kg}\right)}$$

The economic return was calculated as the ratio of income from egg production to the cost of feed (IC). In this economic evaluation, fixed costs such as labor, electricity for lighting and ventilation, and water utilities were maintained as identical variables across all experimental groups, as all birds were housed within the same facility under consistent management. Therefore, the economic efficiency focused on the variable costs directly influenced by feed conversion and egg output under the different lighting and rearing treatments.

Economic efficiency was evaluated using the IC, calculated based on the prevailing local market prices in Thailand during the experimental period (2023). The calculations assumed a standard egg price of 1.0 THB per egg and a feed cost of 18.33 THB per kg. These economic outcomes are intended for comparative purposes within the context of the current study and may vary depending on regional market fluctuations and specific production scales.

### 2.4. Physical Egg Quality Traits

From week 7 to week 20, 20 eggs per replicate were randomly collected weekly for physical egg quality assessment. Each week, eggs collected from each replicate were pooled, and twenty eggs were randomly selected for evaluation. This longitudinal approach enhances statistical power and provides a robust assessment of both internal and external physical egg quality traits. Prior to measurement, eggs were brought to room temperature. The physical traits, including egg weight (EW), albumen height (AH), yolk color (YC), shell thickness (ST), and Haugh unit (HU), were obtained using an EMT-5200 multifunction egg tester (Robotmation, Tokyo, Japan). Yolk color was evaluated using 1 through 15 according to the yolk color chart integrated into the automatic multifunction egg tester, while the HU was automatically computed from the measured AH and EW. Egg shell thickness was measured at four regions of randomly selected eggs (broad end, narrow end, and two equatorial points) using a digital micrometre (Mitutoyo, Tokyo, Japan) with a precision of 0.001 mm, and the values were averaged.

### 2.5. Statistical Analysis

The experiment followed a 3 × 2 factorial arrangement in a completely randomized design, including three LED light colors (red, green, and white) and two rearing systems (cage and floor). The replicate (pen or cage containing 40 birds) served as the experimental unit for all analyses.

Data for overall growth performance (weeks 4–20) were analyzed using a two-way Analysis of Variance (ANOVA). The model included LED color, rearing system, and their interaction as fixed effects. To account for the longitudinal nature of the study, cumulative egg production traits (weeks 6–20), IC (weeks 6–20) and physical egg quality traits (weeks 7–20) were analyzed using a Repeated-measures General Linear Model (GLM). In this model, light color and rearing system were treated as between-subject factors, while time (measurement intervals) was the within-subject factor. To provide a robust mixed-model approach, the replicates were treated as random effects to account for longitudinal dependence. The statistical model is as follows:

$$Y_{ijkl}=\mu +L_{i}+R_{j}+T_{k}+(L\times R{)}_{ij}+(L\times T{)}_{ik}+(R\times T{)}_{jk}+(L\times R\times T{)}_{ijk}+\varepsilon_{ijkl}$$

where: Yijkl is the observed value; μ is the overall mean; L_i_ is the effect of light color; R_j_ is the effect of rearing system; T_k_ is the effect of time interval; the terms in parentheses represent the respective interactions, and ε_ijkl_ is the residual error.

Prior to analysis, all data (including the 20 sampled eggs per replicate for quality traits) were aggregated and averaged at the replicate level to ensure the independence of observations and avoid pseudoreplication. The FCR was calculated starting from the onset of lay (week 6). All analyses were performed using SPSS (Version 29.0). When significant interactions occurred, simple-effects analyses were conducted to explore interval-specific responses and distinguish them from cumulative trends. Mean separation was performed using Tukey’s test, with statistical significance declared at *p* < 0.05.

## 3. Results

### 3.1. Growth Performance

**Figure 1:**
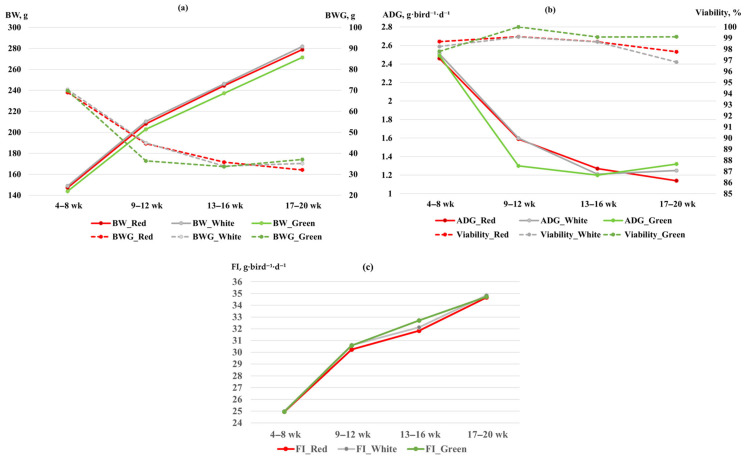
Effect of LED light color (red, white, and green) on (**a**) body weight (BW) and body weight gain (BWG); (**b**) average daily gain (ADG) and viability (%); (**c**) feed intake of laying Japanese quails from 4 to 20 weeks of age. Values are expressed as means for each interval.

The effects of LED light color and rearing system on growth performance were monitored from the initial acclimatization period at 4 weeks through to 20 weeks of age (Table 1). Growth trajectories, including BW, ADG, and feed intake, were illustrated in Figure 1 to provide a comprehensive overview of the birds’ development from the pre-laying phase to peak production. The interaction between LED light color and rearing system was not significant (*p* > 0.05). The effects of LED light color and rearing system on the growth performance of laying Japanese quails during the 4–20 weeks are shown in Table 1. No significant differences were observed among LED light color and rearing system for initial weight (*p* = 0.87 and *p* = 0.91, respectively), BW (*p* = 0.52 and *p* = 0.66), BWG (*p* = 0.45 and *p* = 0.94), ADG (*p* = 0.45 and *p* = 0.94), viability (*p* = 0.15 and *p* = 0.43), or feed intake (*p* = 0.29 and *p* = 0.53).

In addition to the cumulative data, interval-based evaluation was conducted to illustrate the growth performance patterns of quails across different age intervals (Figure 1). The BW and BWG (Figure 1a) increased progressively with advancing age, with the most pronounced rise observed between 9 and 12 weeks, after which growth slowed towards 20 weeks. Birds exposed to red and white light followed similar trajectories, while those under green light displayed slightly lower BWG, particularly during the 9 to 12-week period. In the graph, white and green lines overlap due to nearly identical values, causing the white line to visually mask the green line in BW. The ADG (Figure 1b) peaked at 9 to 12 weeks across all light treatments and subsequently declined as birds approached maturity. Viability remained consistently high (>90%) throughout the entire period, with quails under green light tending to show marginally higher values compared with other treatments. Feed intake (Figure 1c) increased steadily from 4 to 8 and 16 to 20 weeks of age, reflecting the growing nutritional requirements of laying quails. The trends were largely comparable among light color groups, with only minor deviations noted at 12–16 weeks, when green light was associated with slightly higher consumption.

### 3.2. Egg Production

LED light color significantly influenced cumulative egg production parameters from 6 to 20 weeks, including HDP, EM, FCR, and IC (Table 2). Quails exposed to red LED light demonstrated the most favorable egg production performance, with 71.91% HDP rate, an EM of 26.70 $g\cdot {\mathrm{bird}}^{-1}\cdot d^{-1}$, a FCR of 6.11, and an IC of 2.66. In contrast, birds under green light had the poorest outcomes (61.49% HDP, 22.78 g·bird^−1^·d^−1^ EM, 8.83 FCR, and 2.26 income-to-cost), whereas white light produced intermediate values. Neither the rearing system nor its interaction with LED color had a significant effect on these parameters (*p* > 0.05).

**Figure 2:**
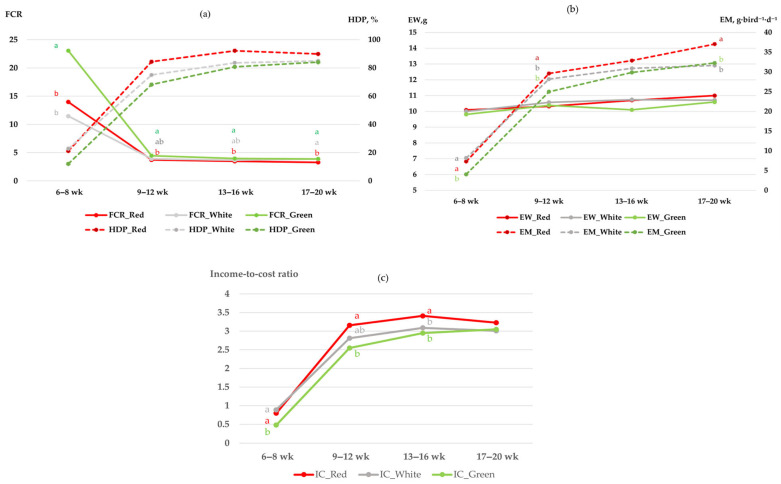
Effects of LED light color (red, white, and green) on (**a**) feed conversion ratio (FCR; kg feed per kg egg mass) and hen-day production (HDP); (**b**) egg weight (EW) and egg mass (EM); (**c**) income-to-cost ratio (IC) of Japanese quails from week 6 to 20. Values represent interval means. Significant interaction effects between LED light color and time were observed for EW, EM, IC and FCR (*p* < 0.05), while HDP was primarily influenced by the main effects of color and time. Different letters within the same age interval indicate significant differences between LED light colors (*p* < 0.05).

In addition to the cumulative outcomes, interval-based data provided further insights into temporal patterns of egg production (Figure 2). Although tracking began earlier, interval-based analysis of these parameters effectively commenced at 6 weeks of age, aligning with the physiological onset of lay. The analysis revealed significant interaction effects between LED light color and time for EW, EM, FCR and IC (*p* < 0.05), indicating that the influence of light spectra varied across different production phases. This interaction highlights that the stimulatory effects of LED colors are not static but are highly dependent on the birds’ physiological maturity and age. Notably, the relatively high FCR values observed during the 6–8 week interval across all treatments (Figure 2a) reflect the physiological transition where birds increase feed intake for reproductive development while egg mass output is still at its initial stage. As shown in Figure 2a, FCR and HDP showed significant variation across light treatments during early and mid-laying stages. Specifically, during the 6 to 8-week interval (representing data from the onset of lay phase), birds under red light achieved significantly higher HDP values (*p* < 0.05) with correspondingly lower FCR, indicating a more efficient conversion of feed into eggs during this critical transition. These advantages were sustained through 9 to 12 weeks, after which the differences among treatments narrowed, with all groups approaching similar HDP values (>80%) and stable FCR from 13 to 20 weeks. The IC (Figure 2c) followed a trajectory similar to egg mass, characterized by a sharp increase as birds progressed from the onset of lay to peak production. During the critical 9 to 12-week interval, red light achieved the highest IC (>3.0), significantly outperforming green light (*p* < 0.05) while white light maintained an intermediate position. This economic advantage for the red light group remained consistent through the mid-to-late laying phases (weeks 13–20), reflecting the sustained egg mass output and efficient feed conversion observed under this spectrum.

**Figure 3:**
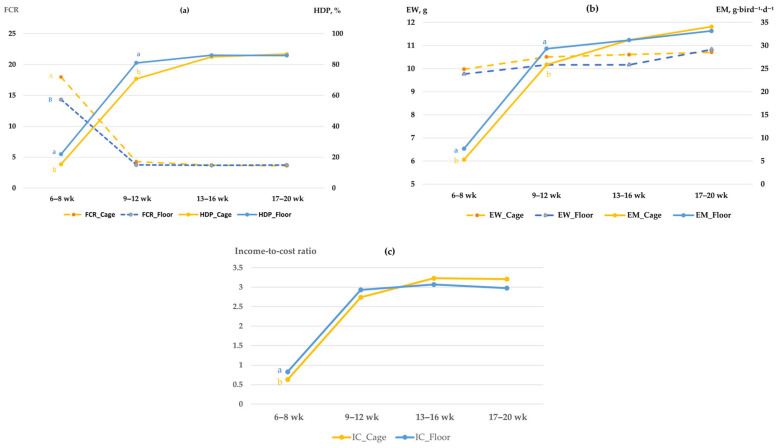
Effects of rearing system (cage and floor) on (**a**) FCR and HDP; (**b**) EW and EM; (**c**) the income-to-cost ratio (IC) of Japanese quails from week 6 to 20. Values represent interval means. Significant interaction effects between rearing system and time were detected for HDP, FCR, EM and IC (*p* < 0.05), whereas EW was influenced by the main effects without significant time-rearing interaction. Different letters within the same age interval indicate significant differences between rearing systems (*p* < 0.05).

Furthermore, significant interaction effects between the rearing system and time were detected for HDP, FCR, EM and IC (*p* < 0.05; Figure 3). Throughout both the initial and early peak laying phases (weeks 6–12), quails in the floor system demonstrated superior production and economic performance compared to those in cages. During the onset of lay (6–8 weeks), the floor system achieved significantly lower FCR (*p* < 0.05; Figure 3a) and a higher IC (*p* < 0.05; Figure 3c). This advantage continued into the early peak production interval (weeks 9–12), where the floor system maintained significantly higher HDP, EM, and IC compared to the cage system (*p* < 0.05; Figure 3a–c). However, these differences diminished as birds reached full physiological maturity from week 13 onwards, with all parameters becoming comparable between the two rearing systems through the remainder of the study. Such temporal interactions underscore the complexity of quail production environments, where the advantages of a specific rearing system may be phase-dependent rather than constant throughout the laying cycle.

The EW and EM (Figure 2b) followed a consistent upward trend with advancing age. Red light produced significantly higher EW at 6 to 8 weeks (*p* < 0.05), whereas both red and white light supported superior EM compared to green light during 9 to 12 weeks (*p* < 0.05). From 13 to 20 weeks, all groups exhibited progressive increases in EW and EM, although red light maintained a slight advantage. Furthermore, the IC (Figure 2c) mirrored the production trends, showing a significant interaction between LED color and time (*p* < 0.05). Notably, red light demonstrated superior economic returns from the very beginning of the laying period, achieving a significantly higher IC compared to green light during the onset of lay (6–8 weeks) and maintaining this leading position through the peak (9–12 weeks) and mid-laying phases (13–16 weeks) (*p* < 0.05). While white light showed intermediate values, it did not surpass the economic efficiency of the red light group during these critical intervals. By the final stage (17–20 weeks), the IC converged across all treatments as the birds reached physiological stability.

### 3.3. Physical Egg Quality Traits

The effects of LED light color and rearing system on cumulative physical egg quality traits of laying Japanese quails from 7 to 20 weeks are presented in Table 3. Neither LED light color nor rearing system had a significant effect on AH (*p* = 0.220 and *p* = 0.203, respectively), ST (*p* = 0.501 and *p* = 0.375), YC (*p* = 0.427 and *p* = 0.682), or HU (*p* = 0.694 and *p* = 0.324). Similarly, no significant interactions between light color and rearing system were observed for all traits (*p* > 0.05).

As shown in Table 3, while the main effects of light color and rearing system were not significant (*p* > 0.05), a significant effect of time (*p* < 0.05) was detected for all parameters, reflecting natural physiological changes associated with the aging of the quails.

**Figure 4:**
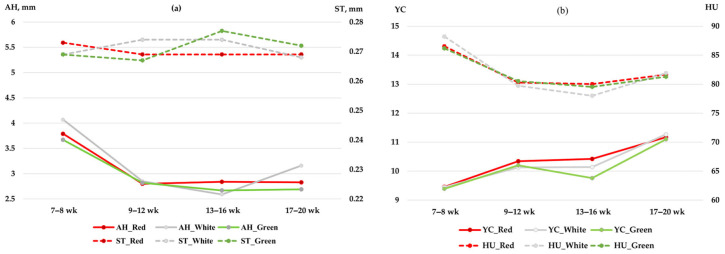
Longitudinal effects of LED light color (red, white, and green) on (**a**) albumen height (AH) and shell thickness (ST); (**b**) yolk color (YC) and Haugh unit (HU) of Japanese quails from week 7 to 20. The data represent temporal variations analyzed through a repeated-measures model. Values are expressed as interval means. Significant effects of time were observed for all traits (*p* < 0.05), while no significant interaction or main effects of light color and rearing system were detected (*p* > 0.05).

To ensure a robust assessment, interval-based evaluation of egg quality was conducted from week 7 onwards (Figure 4). The AH showed a marked decrease between the 7–8 and 9–12 week intervals across all treatments (Figure 4a), followed by a gradual stabilization up to 20 weeks. In contrast, ST remained relatively stable throughout the experimental period, with values consistently maintained around 0.27 mm across all light treatments (Figure 4a). The YC decreased progressively from the 7–8 week interval towards the 13–16 week period under all treatments, with a subsequent slight increase at 17–20 weeks (Figure 4b). Although these changes followed a similar temporal trend, no significant differences were detected between LED colors or rearing systems at any specific interval (*p* > 0.05). Likewise, HU values declined after the initial phase but remained within a high-quality range (>80) toward the end of the study, with all treatments showing parallel trends without significant differences.

## 4. Discussion

Light plays a critical role in regulating circadian rhythms and coordinating essential physiological functions in avian species, including thermoregulation and metabolic activity. Although much of the foundational knowledge on avian photoreception and its endocrine regulation is derived from studies in broiler chickens and laying hens [20,21], comparable photostimulatory mechanisms have also been shown to be associated with reproductive development and overall physiological maturation in Japanese quails [14,15]. This suggests that, despite species-specific differences, the core pathways underlying photostimulation are relatively conserved across domestic poultry.

In this study, neither the color of the LED light nor the rearing system had a significant effect on the growth performance parameters, including final body weight, weight gain, average daily gain, feed intake, or viability. This lack of variation may be attributed to the quails’ physiological adaptability, which allows them to maintain stable growth responses under different lighting conditions, provided that nutritional and environmental management are uniform [15]. Such adaptability may also explain why feed intake remained consistent across treatments, suggesting that visual sensitivity to light colour or rearing system did not alter the quails’ feeding pattern or their ability to perceive and respond to different lighting conditions visually. These findings suggest that Japanese quails exhibited comparable visual sensitivity to the tested LED light colors, regardless of whether they were housed in cages or on floor systems.

The uniformity in feed intake across treatments indicates that neither light spectrum nor rearing system had a discernible impact on the quails’ feeding pattern or their ability to perceive and respond to different lighting conditions visually. These findings are consistent with those of Almeida et al. [19], who reported no significant differences in feed intake, weight gain, or feed conversion ratio in Japanese quails under different LED light colors and are further supported by Al-Hsenawi et al. [28], who similarly observed no significant variation in body weight and weight gain across lighting treatments in quails. Similar observations were reported by Santana et al. [29] and Borille et al. [1], who found no significant differences in feed intake among broiler chickens and laying hens, respectively, when exposed to various LED light colors. Badawi [18] reported no significant differences in average body weight, body weight gain, feed intake, feed conversion ratio, or mortality between Japanese quails reared in battery cages and those reared on floor pens, which aligns with the present study’s findings. Similarly, Padmakumar et al. [30] observed that the average feed efficiency of Japanese quails from 5 to 50 weeks of age was not influenced by the rearing system. In contrast, Razee et al. [31] reported that the rearing system significantly affected body weight gain, feed intake, and feed conversion ratio in growing Japanese quails, with higher performance observed in cage-reared birds. While they conducted their study on mixed-sex populations of quails, the present study focused solely on female quails. This difference in focus may result in varied responses to space availability and environmental conditions. It is well-documented that sex-based differences in growth and behavior exist among poultry species, which may have affected the outcomes of their study [32].

Although the cumulative analysis showed no significant effects of LED light color or rearing system on growth performance, the interval trends provide useful insights. Body weight, weight gain, and ADG increased significantly during the 9-to-12-week period across treatments, reflecting the rapid growth phase before birds reached reproductive maturity. Thereafter, growth velocity declined between 13 and 20 weeks despite continued increases in feed intake, which is consistent with nutrient partitioning toward egg production rather than somatic growth. Similar age-related patterns in quail have been reported, where early growth is rapid and later stages show reduced gains as energy is redirected to reproductive functions [19,28]. Viability remained consistently high across all stages, confirming that neither the lighting spectrum nor the rearing system imposed stress detrimental to survival. These sequential observations complement the cumulative findings by indicating that while overall treatment effects were absent, the 9-to-12-week interval represents the critical window of maximal growth efficiency in Japanese quails. While growth performance was tracked from the commencement of the study (week 4), egg production parameters (Figure 2) were evaluated from week 6 to align with the physiological onset of lay. Furthermore, physical egg quality traits (Figure 4) were reported starting from week 7 to ensure a sufficient and representative sample size of eggs per replicate for robust laboratory analysis.

The present study revealed that LED light color significantly influenced HDP, EM, and FCR, while the rearing system and its interaction with lighting showed no significant overall effects. However, when temporal dynamics were considered, significant interactions between each treatment and time (*p* < 0.05) emerged. This underscores the necessity of evaluating integrated environmental factors rather than viewing them in isolation, as the influence of both light spectra and rearing systems on productivity fluctuated significantly across different stages of the laying cycle. The observed synergy between red LED light and the initial laying phase provides a more comprehensive understanding of quail productivity, suggesting that the physiological response to light is modulated by the specific housing environment over time [3,20]. This finding is consistent with previous reports indicating that the impact of light color and rearing system on productivity fluctuated across different stages of the laying cycle. Quails exposed to red LED light exhibited improved productivity across some production traits, with significantly higher HDP (71.91%) and EM (26.70 $g\cdot {\mathrm{bird}}^{-1}\cdot d^{-1}$). Interestingly, our interval-based analysis revealed that the stimulatory effects of red LED light were most pronounced during the onset of lay and the peak production phases (weeks 6–12). This temporal pattern suggests that quails may exhibit higher sensitivity to longer wavelengths during their initial physiological transition into reproductive maturity [33,34]. As the birds progressed toward the late laying stage (weeks 17–20), the performance gap between light treatments narrowed. This convergence could potentially be attributed to the stabilization of the hypothalamic-pituitary-gonadal axis as the quails reach full maturity, which may diminish the differential impact of light spectra observed during earlier, more dynamic growth phases [33]. While the fundamental principles of avian photoreception are often derived from models in laying hens [20] and broilers [21,35], similar physiological pathways have been observed in Japanese quails [14,15].

Several physiological and metabolic mechanisms described in broader avian literature have been suggested to explain the superior production observed under red light in this study. Red light, with its longer wavelength, is reported to penetrate deeper into avian tissues and is hypothesized to stimulate hypothalamic photoreceptors, which may in turn enhance the secretion of gonadotropin-releasing hormone [21] and downstream reproductive hormones such as luteinizing hormone and follicle-stimulating hormone [20]. Although hormone levels were not directly measured in the present work, the observed production advantages under red light are hypothesized to be mediated by these established hormonal responses. Future investigations incorporating species-specific endocrine profiling for Japanese quails are warranted to confirm these inferred mechanistic pathways. In addition, as noted in general avian models [20,21], red light has been reported to enhance mitochondrial activity and energy metabolism, supporting sustained reproductive output with better feed efficiency. In contrast, green light may favor somatic growth over reproduction [20]; consequently, its influence on reproductive endocrine pathways in quails appears to be comparatively weaker, which aligns with the reduced egg production and efficiency observed in the present study. White light, combining multiple wavelengths, provided intermediate effects by balancing growth-related and reproductive responses, a trend supported by comprehensive reviews of light-spectrum effects in poultry [35].

The FCR was significantly affected by LED color, red light (6.11) and white light (5.72), yielding better feed efficiency compared to green light (8.83, *p* < 0.01). This efficiency under red lighting corroborates the findings of Molino et al. [36], who reported that Japanese quails reared under red LED lighting exhibited better feed conversion ratios per dozen eggs compared to other colors, likely due to the light’s ability to modulate feeding behavior and metabolic activity that minimizes energy wastage. Similar conclusions were reached by Furtado et al. [37] and Ahmad et al. [25], who emphasized that lighting strategies enhancing both productivity and feed efficiency ultimately improve profitability in quail production. The economic analysis in the present study further supports this, as evidenced by the significantly higher IC maintained under red light from the onset of lay through the mid-laying phase (weeks 6–16; Figure 2c). This indicates that the enhanced reproductive stimulation under the red spectrum translates directly into sustained financial returns, outweighing the minor numerical advantages in feed efficiency seen under white light. Furthermore, the temporal interaction observed in rearing systems reveals that the floor system provided a more economically favorable environment, particularly during the onset of lay (6–8 weeks), where it achieved a significantly higher IC and lower FCR compared to the cage system (Figure 3a,c). This initial advantage in profitability and feed efficiency suggests that floor-reared quails possess a better adaptive capacity during the first 8 weeks of production. While the floor system maintained higher HDP through the early peak phase (6–12 weeks), the economic and efficiency gaps between the two systems gradually diminished after week 12, as both environments reached comparable performance levels during full maturity.

Huber-Eicher et al. [38] reported that exposure to red light significantly influenced sexual maturation in laying hens and attributed this response to the light’s specific wavelength rather than its intensity. Overall, red light has been shown to be more effective in stimulating egg production compared with green or blue light, which exerts little to no impact. In commercial layer flocks, egg production during the first and second laying cycles was strongly affected by light color, with the highest egg output observed under red light treatment [25]. Although EW was not significantly influenced by LED color or rearing system, the consistently moderate values observed across treatments indicate stability in production performance. Similar trends were reported by Nasr et al. [39], who found that dietary and lighting manipulations often impact production rate more than individual egg traits such as weight.

Regarding the rearing system, egg production did not differ significantly between rearing systems. This result aligns with the findings of Soares et al. [40], who investigated welfare indicators in laying Japanese quails and reported that although production differences between cage and floor systems were not always significant, floor housing enhanced welfare by supporting natural behaviors. However, the significant interaction between rearing system and time (*p* < 0.05) observed in our study revealed that cage-reared quails achieved higher HDP and EM specifically during the 9–12 week interval. This temporal advantage may be attributed to a more stabilized environment and reduced competition for resources within the cage system during the peak production phase, allowing for more efficient energy allocation toward egg formation. These observations are consistent with the recent study by Hossain et al. [27], who suggested that housing effects on quail performance can become more evident during periods of high physiological demand. When production traits were examined across the laying intervals, clear differences emerged that complemented the cumulative findings. The HDP and EM steadily increased with advancing age, with the most notable improvements occurring between 9 and 12 weeks and stabilizing thereafter. This trend reflects the natural progression from sexual maturity into peak laying, consistent with reports by Nelson et al. [41] and Molino et al. [36], who noted that quail egg production generally rises sharply after the onset of lay before plateauing at maturity. This phase (8–12 weeks) also marks a physiological transition where nutrient partitioning shifts from somatic growth towards intensive egg production, explaining why the maximal growth rate observed in the same period begins to plateau as reproductive output reaches its peak. The FCR also improved as the birds matured, particularly from 13 to 16 weeks, when higher egg output was achieved with proportionally lower feed intake. This efficiency gain may be linked to enhanced ovarian activity and more stable energy partitioning toward egg formation, as described by Furtado et al. [37] in quails and Huber-Eicher et al. [38] in laying hens. The superior performance observed under red LED lighting across these intervals suggests that red light may accelerate the attainment of peak production, supporting earlier hormonal stimulation of follicular development and ovulation, as previously reported by Bobadilla-Mendez et al. [15]. Egg weight remained relatively stable across weeks, which agrees with cumulative results showing no significant influence of light color or rearing system. This stability indicates that while laying rate and efficiency were strongly modulated by light spectrum, egg weight was largely unaffected, as also observed by Nasr et al. [39]. Together, these weekly distributions illustrate that the beneficial effects of red LED light on productivity were not only cumulative but also evident at critical phases of the laying cycle, particularly during 9 to 12 and 13 to 16 weeks, when birds reached and sustained peak laying performance. The absence of a significant interaction between lighting and rearing system indicates that LED light color exerted a dominant independent effect on production traits.

Furthermore, the consistent production performance observed in this study suggests that the experimental conditions provided a suitable environment for the quails. It is important to note that throughout the 20-week period, no incidences of abnormal behaviors, such as severe feather pecking or cannibalism, were observed across any of the light treatments or rearing systems. This indicates that the 40-lux intensity and the specific LED spectra used did not induce detrimental stress levels that typically lead to such behavioral issues. This observation aligns with the high viability rate (>95%) recorded in our study, further supporting the conclusion that both cage and floor systems under the tested LED colors maintained the quality of bird functioning and welfare.

The current study found that LED light color and rearing systems did not significantly influence cumulative physical egg quality, including AH, YC, ST, and HU. This lack of variation may be attributed to the birds’ physiological adaptability to different light spectra, as well as consistent nutritional and environmental management across treatments. However, the repeated-measures analysis revealed a significant effect of time on all egg quality parameters (*p* < 0.05), regardless of the light or rearing treatments. This temporal shift, particularly the decline in AH and HU values observed as the birds matured, is likely a manifestation of the natural aging process in quails. The significance of this temporal decline was further validated by our repeated-measures analysis, which identified time as a dominant factor (*p* < 0.05) influencing internal quality throughout the laying cycle, even when cumulative treatment effects were not significant (Table 2). These findings align with recent observations by Hossain et al. [27] and Abuoghaba et al. [42], who noted that while external factors like lighting may not alter egg quality, the bird’s age remains a dominant factor influencing internal quality traits throughout the laying cycle. The AH was slightly higher under white light, possibly reflecting subtle improvements in protein deposition and albumen synthesis, whereas yolk color showed marginally higher values under red light, which may relate to enhanced carotenoid metabolism [15,42]. Nevertheless, the differences were not statistically significant. This finding aligns with Raziq et al. [20] and Nunes et al. [14], who reported that internal egg quality traits such as HU and YI were not significantly affected by LED light in laying quail. Similarly, Furtado et al. [37] and Soares et al. [40] highlighted the inherent capacity of quails to maintain stable egg quality traits under varying housing and lighting conditions, provided that nutrition and management are optimized.

When physical egg quality traits were evaluated across weeks, the distributions further confirmed the stability observed in the cumulative data. While AH and HU showed a general decline consistent with the aging effect observed in the repeated-measures analysis, ST remained relatively consistent throughout the laying period, with only minor fluctuations across intervals. These results suggest that protein deposition in albumen and calcium mobilization for shell formation were not markedly influenced by light spectrum or rearing system, but rather maintained by the birds’ inherent physiological regulation, as similarly reported by Nunes et al. [14] in quails and Raziq et al. [20] in laying hens. The YC and HU also showed no distinct weekly improvements, reinforcing the notion that these traits are less sensitive to photo stimulation compared to egg production parameters. Our findings confirmed that YC was not significantly influenced by LED light color or rearing system throughout the study. This aligns with the conclusions of Furtado et al. [37] and Soares et al. [40], suggesting that light environment plays a limited role in modulating egg pigmentation when dietary composition and management are consistent. In general, the weekly interval analysis confirmed the cumulative findings: while egg production traits responded significantly to LED light color, physical egg quality traits such as AH, ST, YC, and HU remained stable across the laying cycle. This stability highlights the resilience of Japanese quail egg quality against external environmental factors, provided optimal feeding and husbandry conditions are ensured.

While these findings highlight the benefits of red LED light for improving production performance and profitability in laying Japanese quails, the underlying physiological and hormonal mechanisms were not investigated. Moreover, the study focused primarily on physical egg quality parameters. Future studies should therefore explore metabolic, endocrine, and direct stress-related biomarkers (e.g., corticosterone levels) alongside nutritional egg quality traits and edible portion indices to address current research gaps concerning the long-term impact of light spectra on both bird functioning and comprehensive egg quality.

## 5. Conclusions

In conclusion, LED light color is a key factor in improving the productivity and economic returns of laying Japanese quails, while the rearing system has a limited cumulative impact. Red LED light significantly enhanced EP, FCR, and IC, particularly from the onset of lay through the mid-laying phase (weeks 6–16), making it a practical choice for optimizing reproductive performance. While both cage and floor systems remain viable, the floor system offers distinct benefits in feed efficiency and profitability during the initial production stage (weeks 6–12). Although physical egg quality remained stable across different lighting and housing conditions, it was significantly influenced by the natural aging process. The interaction effects confirm that red light independently stimulates the reproductive axis, while both cage and floor systems remain viable management options for commercial quail production.

## Figures and Tables

**Table 1:** Effect of LED light color (red, white, and green) and rearing system (laying cage and floor rearing) on production performance of laying Japanese quails during the 4 to 20 weeks.

Trait	LED Color				*p*-Value	Rearing System			*p*-Value
	Red	White	Green	SEM	LED Color	Cage	Floor	SEM	Rearing System
Initial weight (g)	108.19	108.04	108.09	0.18	0.87	108.09	108.12	0.16	0.91
Final body weight (g)	291.61	293.74	289.37	4.51	0.52	291.75	289.39	3.73	0.66
^1^ BWG (g)	183.72	184.39	177.01	4.43	0.45	181.92	181.50	3.69	0.94
^2^ ADG (g·bird^−1^·d^−1^)	1.64	1.65	1.58	0.07	0.46	1.62	1.62	0.03	0.94
Viability (%)	95.14	95.56	97.33	0.76	0.15	96.41	95.61	0.69	0.43
^3^ FI (g·bird^−1^·d^−1^)	30.42	30.64	30.74	0.13	0.30	30.55	30.66	0.12	0.53

^1^ BWG, body weight gain; ^2^ ADG, average daily gain; ^3^ FI, feed intake.

**Table 2:** Overall effects of LED light color and rearing system on the production performance and economic efficiency of laying Japanese quails (weeks 6–20) based on repeated-measures analysis.

Trait	LED Color					Rearing System			
	Red	White	Green	SEM	*p*-Value	Cage	Floor	SEM	*p*-Value
Hen-day production (%)	71.91 ^a^	66.37 ^ab^	61.49 ^b^	2.01	0.01	64.47	68.72	1.64	0.09
Egg weight (g)	10.53 ^a^	10.51 ^a^	10.22 ^b^	0.08	0.04	10.45	10.38	0.07	0.49
Egg mass (g·bird^−1^·d^−1^)	26.70 ^a^	24.71 ^ab^	22.78 ^b^	0.73	<0.01	24.12	25.35	0.60	0.17
^1^ FCR	6.11 ^b^	5.72 ^b^	8.83 ^a^	0.43	<0.01	7.39	6.37	0.35	0.06
Income-to-cost ratio	2.66 ^a^	2.45 ^ab^	2.26 ^b^	0.08	<0.01	2.46	2.46	0.06	0.10

^a,b^ Values within a row with different superscripts differ significantly. ^1^ Feed conversion ratio (kg feed per kg egg mass). Note: Significant interaction effects (*p* < 0.05) between light color × time and rearing system × time were detected for these traits; detailed temporal patterns are illustrated in Figure 2 and Figure 3.

**Table 3:** Overall effects of LED light color and rearing system on physical egg quality traits of Japanese quails (weeks 7–20) based on repeated-measures analysis.

Trait	LED Color					Rearing System			
	Red	White	Green	SEM	*p*-Value	Cage	Floor	SEM	*p*-Value
Albumen height (mm)	3.07	3.17	2.97	0.08	0.24	3.01	3.13	0.06	0.21
Egg shell thickness (mm)	0.27	0.27	0.27	0.00	0.49	0.27	0.27	0.00	0.26
Egg yolk colour	10.36	10.25	10.12	0.14	0.49	10.27	10.21	0.11	0.70
Haugh unit	81.94	81.90	81.29	0.48	0.58	81.52	81.90	0.39	0.52

Note: No significant interactions were observed between light color, rearing system, and time for any egg quality traits. While the main effects of light color and rearing system were also not significant (*p* > 0.05), a significant effect of time (*p* < 0.05) was detected for all parameters, reflecting the natural physiological changes associated with the aging of the quails.

## Data Availability

The original contributions presented in this study are included in the article. Further inquiries can be directed to the corresponding author.

## Abbreviations

The following abbreviations are used in this manuscript:

LED	Light-emitting diode
HDP	hen-day egg production
EW	egg weight
EM	egg mass
FCR	feed conversion ratio per kilogram of egg produced
AH	albumen height
ST	shell thickness
YC	yolk color
HU	Haugh unit
